# Informal Caregivers in Amyotrophic Lateral Sclerosis: A Multi-Centre, Exploratory Study of Burden and Difficulties

**DOI:** 10.3390/brainsci11081094

**Published:** 2021-08-20

**Authors:** Éilís Conroy, Polly Kennedy, Mark Heverin, Iracema Leroi, Emily Mayberry, Anita Beelen, Theocharis Stavroulakis, Leonard H. van den Berg, Christopher J. McDermott, Orla Hardiman, Miriam Galvin

**Affiliations:** 1 Academic Unit of Neurology, School of Medicine, Trinity College Dublin, D02 PN40 Dublin, Ireland; conroyei@tcd.ie (É.C.); pokenned@tcd.ie (P.K.); mark.heverin@tcd.ie (M.H.); hardimao@tcd.ie (O.H.); 2 Department of Psychiatry, St. James’ Hospital, Global Brain Health Institute, Trinity College Dublin, D08 PR2A Dublin, Ireland; iracema.leroi@tcd.ie; 3 Department of Psychological Services, Sheffield Teaching Hospitals NHS Foundation Trust, Sheffield S10 2JF, UK; emily.mayberry@sheffield.ac.uk; 4 Department of Neuroscience, The University of Sheffield, Sheffield S10 2HQ, UK; t.stavroulakis@sheffield.ac.uk (T.S.); c.j.mcdermott@sheffield.ac.uk (C.J.M.); 5 Centre of Excellence for Rehabilitation Medicine, UMC Utrecht Brain Centre, University Medical Centre Utrecht, 3584 CX Utrecht, The Netherlands; j.a.j.beelen@umcutrecht.nl; 6 De Hoogstraat Rehabilitation, 3584 CX Utrecht, The Netherlands; 7 UMC Utrecht Brain Centre, Department of Neurology, University Medical Centre Utrecht, 3584 CX Utrecht, The Netherlands; l.h.vandenberg@umcutrecht.nl; 8 National Neuroscience Centre, Department of Neurology, Beaumont Hospital, D09 V2N0 Dublin, Ireland

**Keywords:** amyotrophic lateral sclerosis (ALS), motor neuron disease (MND), informal caregivers, national, burden, emotion, distress, mixed-methods

## Abstract

Amyotrophic lateral sclerosis (ALS)/motor neuron disease (MND) is a systemic and fatal neurodegenerative condition for which there is currently no cure. Informal caregivers play a vital role in supporting the person with ALS, and it is essential to support their wellbeing. This multi-centre, mixed methods descriptive exploratory study describes the complexity of burden and self-defined difficulties as described by the caregivers themselves. Quantitative and qualitative data were collected during face-to-face interviews with informal caregivers from centres in the Netherlands, England, and Ireland. Standardised measures assessed burden, quality of life, and psychological distress; furthermore, an open-ended question was asked about difficult aspects of caregiving. Most caregivers were female, spouse/partners, and lived with the person with ALS for whom they provided care. Significant differences between national cohorts were identified for burden, quality of life, and anxiety. Among the difficulties described were the practical issues associated with the caregiver role and emotional factors such as witnessing a patient’s health decline, relationship change, and their own distress. The mixed-methods approach allows for a more nuanced understanding of the burden and difficulties experienced. It is important to generate an evidence base to support the psychosocial wellbeing and brain health of informal caregivers.

## 1. Introduction

Amyotrophic lateral sclerosis (ALS), a sub-type of motor neuron disease (MND), is a progressive, neurodegenerative disease, which impacts the physical, communicative, and cognitive functioning of those affected. There is currently limited treatment, and for the majority of patients, death occurs within three years of symptom onset [1]. Management of ALS is palliative; treatment consists of symptom management and is aimed at maximising quality of life and minimising the burden of disease for patients and caregivers [2].

For people with ALS, a considerable amount of care is provided by family and friends, with informal caregivers playing a significant role in the care ecosystem [3]. Informal caregivers are key figures in the provision of care [3], which may allow the person with ALS to be cared for in their own home [4]. Caring for a partner or family member with a progressive neurological illness has been recognised as being a source of burden and distress, resulting in lowered levels of quality of life [5,6]. Caring for someone with ALS affects the physical, psychological, and emotional wellbeing of the caregiver [7,8]. High levels of burden have been identified [4,9,10] among informal caregivers in ALS, and they can spend over 100 hours per week providing care [11].

The aim of this paper is to characterise and describe informal ALS caregiver cohorts attending three ALS multidisciplinary clinical centres (Dublin, Ireland; Utrecht, The Netherlands; and Sheffield, England) and describe the self-reported burden and difficulties associated with caregiving. The incidence of ALS in Ireland is 3.1 per 100,000, and the prevalence is approximately 8 per 100,000 [12]. In the Netherlands, the prevalence of ALS is 9.5 per 100,000 persons [3] and 8.58 per 100,000 in the UK [13]. At least 80% of all ALS patients within Ireland and the Netherlands attend the National ALS/MND Clinic in Beaumont Hospital, Dublin [14] and University Medical Centre Utrecht, respectively [15]. The catchment area of the Sheffield Motor Neuron Disease Care Centre & Clinical Neuropsychology Services in Sheffield Teaching Hospitals NHS Trust is approximately four million (similar to Utrecht and Dublin).

These countries vary in terms of culture, population characteristics, health care systems, and economies. Macro-level characteristics such as these are not part of this analysis. A description of the differences and similarities in these three cohorts will contribute to better-informed interventions to address the needs of caregivers.

## 2. Methodology

### 2.1. Participants

As part of a European multi-centre study of people with ALS (A Programme for Amyotrophic Lateral Sclerosis Care in Europe [16]), the primary informal caregivers of patients attending specialist ALS clinics in Beaumont Hospital, Dublin (Ireland), University Medical Centre Utrecht (The Netherlands), and Sheffield Teaching Hospitals NHS Trust (England) were recruited to participate in a study of their own wellbeing. Caregivers of all patients who attended clinics during a 12-month period, and were identified by the person with ALS as their main informal caregiver, were invited to participate in a research interview about their experiences. They were provided with information about the research and given time to consider their participation. Formal paid caregivers and people aged less than 18 years were not recruited for the study. These caregivers were providing care for people with ALS at all stages along the disease trajectory, both incidence and prevalence cases of ALS.

**Figure 1:**
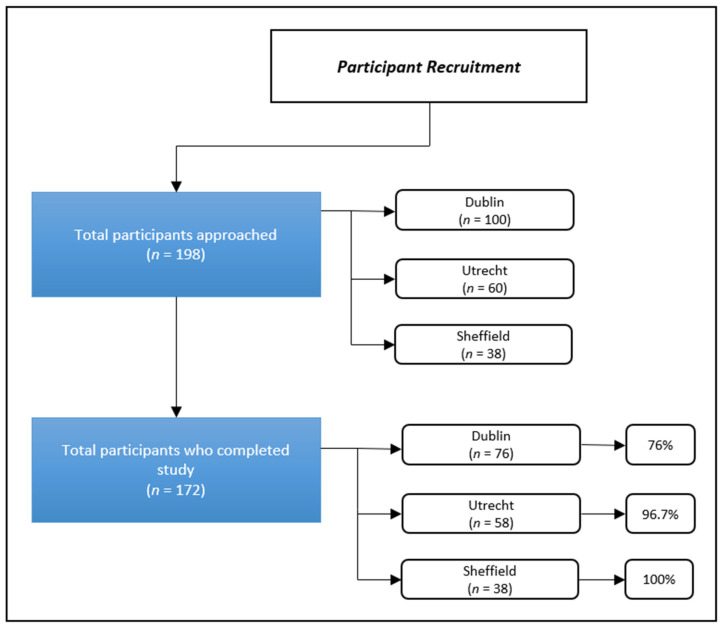
Participant recruitment. Participants were included in this analysis if they completed demographic data, at least one measure, and the open-ended question.

Eighty-two caregivers were recruited in the ALS clinic in Dublin, and 76 agreed to participate, 60 recruited from University Medical Centre Utrecht with 58 agreeing to participate and of the 39 people recruited from the Sheffield Clinic, 38 participated in the study (see Figure 1 for details—participants recruited and percentage of those included in the analysis).

Pilot-tested semi-structured interviews were conducted in the caregiver’s own home at a time convenient for them by a member of the research team. The confidentiality of the research process and the anonymity of their responses were both assured. Ethical approval was received from the Beaumont Hospital Ethics (Medical Research) Committee (REC/REF 12/84), the Research Ethics Committee, the Medical Ethics Committee of the University Medical Centre Utrecht (ethics approval code 15−708; national code NL56609.041.16) and National Research Ethics Service Committee Yorkshire and the Humber—Bradford Leeds (REC/REF 15/YH/0014).

### 2.2. Measures

Demographic and socio-economic details were collected, as well as a series of standardised measures commonly used in ALS research [9,17] to assess caregiver burden, psychological distress, and quality of life. In an open-ended question, caregivers were asked to describe what, for them, are some difficult things about caregiving (wording shown below).

#### 2.2.1. Psychological Distress

The hospital anxiety and depression scale (HADS) [18] is a statistically reliable measure composed of two subscales detecting depression (HADS-D) and anxiety (HADS-A). The use of a summed HADS total score (HADS-T) is seen as an adequate estimate of general psychological distress [19]. A cut-off score of ≥12 was used to identify those with probable psychological distress [20].

#### 2.2.2. Quality of Life

Quality of life (QoL) was assessed using the quality of life in life-threatening illness—family carer version 2 (QOLLTI-F v2; [21]). The QOLLTI-F Total score is an average of seven subscale scores (environment, patient condition, carer’s own state, carer’s outlook, quality of care, relationships, and financial worries). In addition, this measure includes a single item constructed to measure self-reported QoL and is referred to as the global score. All items are numerically rated (scale: 0–10) based on a two-day time frame, with higher scores being indicative of greater QoL. In this analysis, the Total score was used as a measure of self-assessed QoL.

#### 2.2.3. Burden

The Zarit burden inventory (ZBI; [22]) is a widely used instrument for measuring subjectively assessed caregiver burden. It has 22 items rated on a 0–4 scale, with a maximum score of 88. Total scores ≥ 24 indicate high levels of burden, and scores < 24 indicate low levels of burden [23].

#### 2.2.4. Open-Ended Question

As part of the semi-structured interview, caregivers were asked, “*For you, what are some things that are difficult about caregiving?*”

### 2.3. Data Analysis

A mixed-methods approach was used for the purposes of complementarity and facilitating additional coverage [24] to get a more comprehensive picture of the burden and experiences associated with the informal caregiving of ALS. The analysis is based on the responses from 172 informal caregivers in Ireland (*n* = 76), Netherlands (*n* = 58) and England (*n* = 38) (see Figure 1).

#### 2.3.1. Statistical Analysis

Descriptive statistics summarised the socio-demographic and wellbeing measures of caregivers in Ireland, England, and the Netherlands. These are presented as percentage (%), mean with standard deviation (SD), or median with interquartile range (IQR), as relevant. The normal Gaussian data distribution was tested using the Shapiro–Wilk test and the Kolmogorov-Smirnov test. Kruskal-Wallis H tests, Fisher’s Exact tests, Chi-Square tests, and One-way ANOVAs with Tukey, Games-Howell, or two proportion z-test post-hoc analyses were used to explore the mean/median differences in characteristics and wellbeing measures across the three caregiver cohorts. Statistical analyses were carried out using IBM^®^ Statistical Package for the Social Sciences (SPSS^®^) [25] version 26.

#### 2.3.2. Qualitative Analysis

The ‘codebook’ approach to thematic analysis was used to identify, analyse, and report themes from caregiver responses [26]. Coding was carried out by two coders (ÉC and PK). An inductive approach to coding was driven by the content of the data, with both descriptive and interpretative approaches used during theme development and refinement [27]. The Irish text data were coded, and a coding frame was generated. This coding frame was then applied to the Dutch (translated to English) and English data, respectively and the frame was expanded and refined to include codes and themes derived from the process. There was discussion on points of agreement/disagreement leading to consensual validation [28]. The codes generated and themes constructed in this analysis were reviewed, and the credibility of the findings was established based on clinical experience. Microsoft Excel v16.49 (2021) was used to collate and manage the qualitative data, audit record coding patterns, and theme development.

## 3. Results

### 3.1. Caregiver Characteristics: Descriptive Statistical Analysis

Caregiver characteristics are described for each country separately and presented in Table 1. There were some differences in demographic variables for the three caregiver cohorts in terms of age (F(2, 82.55) = 8.828, *p* < 0.0005), and health status (Fisher’s exact test = 21.651, *p* = 0.003). Games-Howell post hoc analysis revealed the difference between the mean age of caregivers in Ireland and the Netherlands (8.371, 95% CI (3.096, 13.646)) was statistically significant (*p* = 0.001).

There were also differences in the number of hours of care provided per week (χ^2^(2) = 21.216, *p* < 0.0005). Dunn’s (1964) post hoc analysis with a Bonferroni correction revealed a statistically significant difference in hours of care between the Irish and Dutch cohorts (*p* = 0.000). No other differences in caregiver characteristics were found.

#### 3.1.1. Caregivers—ALS Centre Dublin

The majority of the Irish caregiver cohort was female (73.7%) and family (97.3%). Eighty-eight per cent lived with the person with ALS for whom they provided care. The mean age was 57 years, ranging from 27 to 81 years. An average of 35.11 h of care was provided each week (median 9.5 h). Eighty-five per cent rated their own health as either excellent, very good, or good, while 40% said they also had long-term health problems. Forty-three per cent were employed at the time of their interview.

#### 3.1.2. Caregivers—ALS Centre Utrecht

Dutch caregivers in this study were also predominantly female (61.8%) and family (96.3%). Eighty-nine per cent lived with the person with ALS for whom they provided care. The mean age was 65 years, ranging from 45 to 80 years. Caregivers spent an average of 96.86 h a week providing care (median 142.5 h). Eighty-five per cent self-rated their own health as excellent, very good, or good, while 34.5% said they also had long-term health problems at the time of the interview. Approximately 33% were in employment.

#### 3.1.3. Caregivers—ALS Centre Sheffield

The majority of caregivers in England were female (81.6%) and family (97.4%). Eighty-nine per cent lived with the person with ALS for whom they provided care. The mean age of the caregiver was 60 years, ranging from 40 to 74 years. Caregivers spent an average of 71 h per week providing care (median 30 h). Eighty-four per cent self-rated their own health as excellent, very good, or good, while 56.8% said they also had long-term health problems at the time of the interview. Approximately 32% were in employment.

### 3.2. Wellbeing Measures

The results from the analyses of outcome measures are presented in Table 2.

#### 3.2.1. Wellbeing Outcomes Overview

The mean caregiver burden score for the Irish cohort was 14.7, and 21% were categorised as highly burdened. The average psychological distress score (HADS-T) was 12.74, with 46% scoring above the cut-off for ‘probable’ clinical levels of distress. On a 0–10 scale, QoL was relatively high, with a mean score of 7.22.

The mean burden score recorded in the Dutch cohort was 23.17, with almost half of respondents in the high burden category. On the HADS-T measure, 31% reached the cut-off score for probable clinical levels of psychological distress. The QoL mean score for the Dutch caregivers was 6.51.

The English caregivers had a mean burden score of 24.47, with 50% in the high burden category. The HADS-T mean score was 11.88, with approximately 24% considered at levels of probable psychological distress. QoL mean score was 6.91 for caregivers in Sheffield.

Some statistically significant differences between the cohorts were identified for burden, anxiety, and QoL. These are explored in more detail in the next section.

#### 3.2.2. Burden Comparison

**Figure 2:**
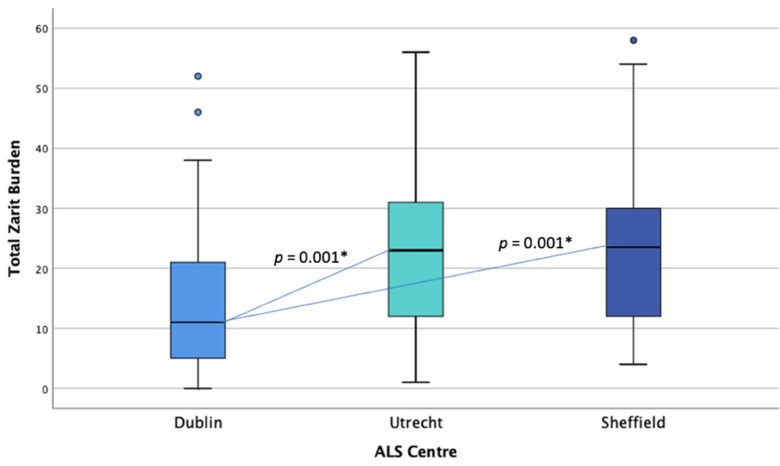
Distribution of burden (ZBI) scores across centres. * Statistical significance.

A one-way ANOVA was conducted to examine differences in caregiver ZBI scores between countries. The data were normally distributed for Sheffield and Utrecht but not for Dublin (see Figure 2). There was homogeneity of variances, as assessed by Levene’s test of homogeneity of variances (*p* = 0.155). ZBI scores were statistically significantly different between countries, *F*(2, 155) = 9.813, *p* < 0.001. ZBI score was lower in the Irish (*M* = 14.70, *SD* = 11.276) than the Dutch (*M* = 23.17, *SD* = 13.57), and English cohorts (*M* = 24.47, *SD* = 14.21). Tukey’s post-hoc analysis revealed that the mean difference between the Irish and Dutch burden levels (−8.466, 95% CI (−13.94, −2.99)) was statistically significant (*p* = 0.001), as well as between the Irish and English burden levels (−9.766, 95% CI (−16.05, −3.48), *p* = 0.001). No statistically significant difference was found between Dutch and English caregiver burden scores. Chi-square analysis showed significant differences between centres for high and low burden categorization, χ^2^(2, *n* = 158) = 13.48, *p* = 0.001. Post-hoc analysis with pairwise comparisons using the z-test of two proportions with a Bonferroni correction showed the Dublin cohort of caregivers to be significantly different compared to the other centres, with fewer caregivers in the high burden category, *p* < 0.05. No significant difference was found between Sheffield and Utrecht in terms of burden categorisation.

#### 3.2.3. Psychological Distress Comparison

**Figure 3:**
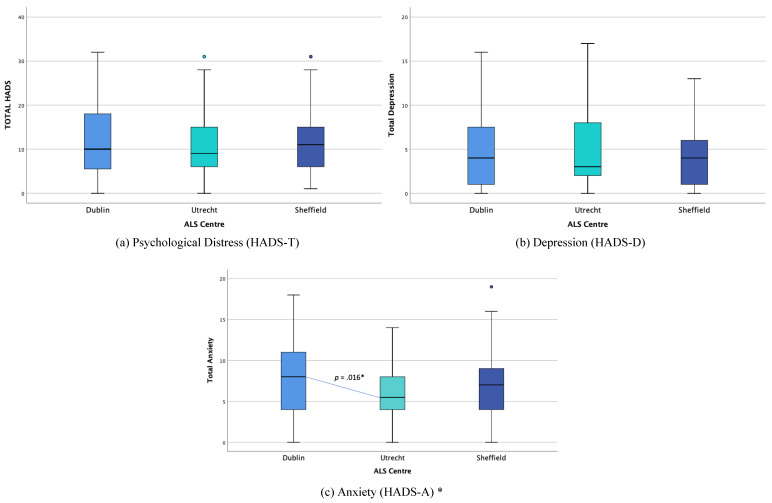
Distribution of psychological distress (HADS) scores across centres. * Statistical significance.

Kruskal-Wallis H tests were conducted to determine if there were differences in HADS-T and HADS-D scores between caregiver countries. The data were not normally distributed for each group (see Figure 3). HADS-T scores were lower than for the Netherlands (*Mdn* = 9), than Ireland (*Mdn* = 10), or England (*Mdn* = 11), but the differences were not statistically significant, χ^2^(2) = 0.997, *p* = 0.607. HADS-D scores were slightly lower in the Netherlands (*Mdn* = 3) than Ireland and England (*Mdn* = 4), but the differences were not statistically significant, χ^2^(2) = 0.302, *p* = 0.860.

A one-way Welch ANOVA was conducted to determine if HADS-A scores were different for caregivers between countries. The data were normally distributed for each group, but the homogeneity of variances was violated, as assessed by Levene’s Test of Homogeneity of Variance, based on means (*p* = 0.043). HADS-A scores were statistically significantly different between countries, Welch’s *F*(2, 83.176) = 4.266, *p* = 0.017. HADS-A scores were lower in the Dutch (*M* = 5.85, *SD* = 3.40) cohort than the English (*M* = 7.44, *SD* = 4.63) and Irish cohorts (*M* = 7.51, *SD* = 1.24). Games-Howell post hoc analysis revealed that the mean increase from the Dutch to the Irish cohort (−2.009, 95% CI (−3.71, −0.31)) was statistically significant (*p* = 0.016). Chi-square analysis found no significant differences between centres for caregivers in the “probable distress” category, χ^2^(2, *n* = 158) = 0.586, *p* = 0.746.

#### 3.2.4. Quality of Life Comparison

**Figure 4:**
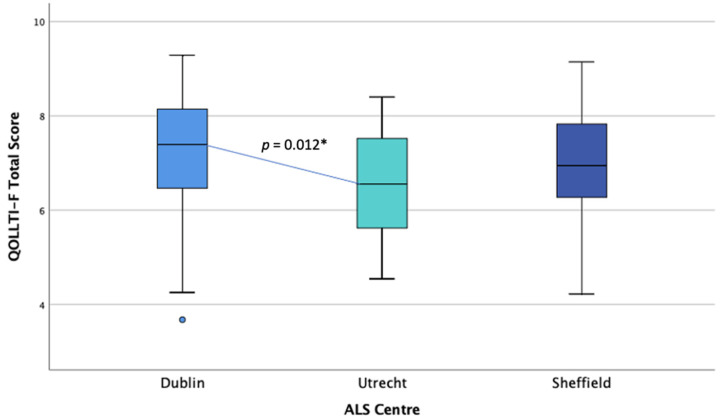
Distribution of quality of life (QOLLTI-F) scores across centres. * Statistical significance.

A one-way ANOVA was conducted to examine differences in caregiver QoL (QOLLTI-F) scores between countries. The data were normally distributed for England and the Netherlands, but not for Ireland, as assessed by a boxplot and Shapiro-Wilk test (*p* > 0.05) (see Figure 4). There was homogeneity of variances, as assessed by Levene’s test of homogeneity of variances, based on means (*p* = 0.369). QOLLTI-F scores were statistically significantly different between countries, *F*(2, 144) = 4.233, *p* = 0.016. QoL was lowest in the Dutch cohort (*M* = 6.51, *SD* = 1.08) and highest in the Irish cohorts (*M* = 7.22, *SD* = 1.38), with the English score in between (*M* = 6.91, *SD* = 1.27). Tukey’s post-hoc analysis revealed that the mean difference between the Dutch and Irish QoL levels (−0.708, 95% CI (−1.28, −0.13)) was statistically significant (*p* = 0.012).

### 3.3. Qualitative Analysis

*“For you, what are some things that are difficult about caregiving?”* The text responses to this open-ended question were analysed qualitatively.

Over one-third of caregivers in Dublin and Utrecht and 13% in Sheffield indicated that there was nothing difficult about caregiving or that there was not anything difficult about it ‘*at the moment’* and ‘*they are coping’*.

**Figure 5:**
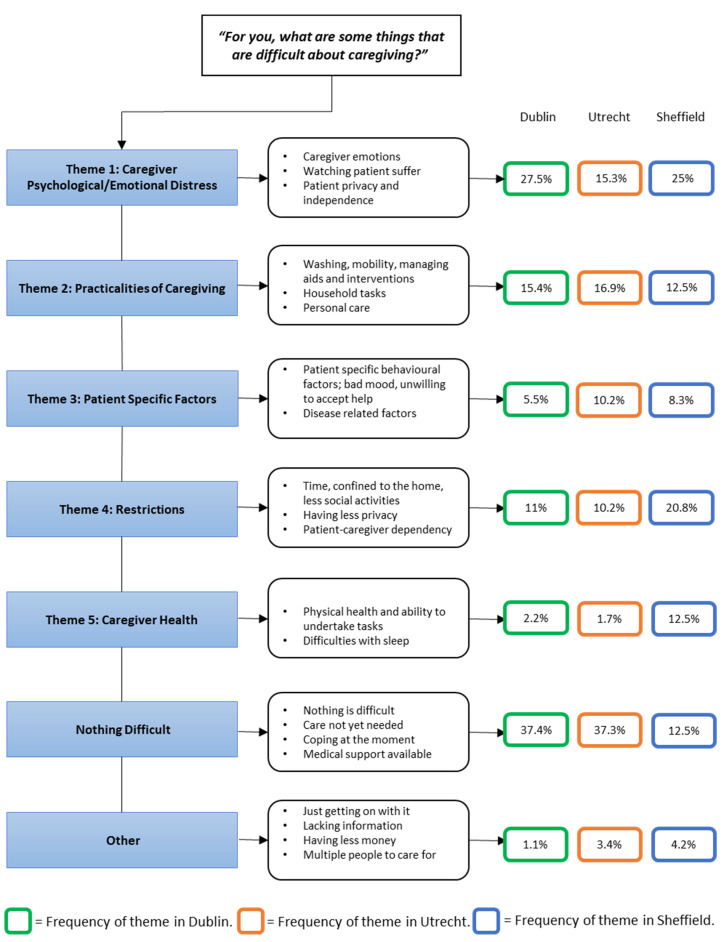
Qualitative Themes and Frequencies. Frequency of themes mentioned by caregivers in each centre ^a^. ^a^ The frequency represents the percentage of responses coded as that theme, as a percentage of all coded responses at that centre. A response could be coded to more than one theme.

For those respondents who indicated specific difficulties associated with caregiving, five main themes and composite subthemes were generated from the coded responses (Figure 5): ‘Caregiver Psychological/Emotional Distress’, ‘Practicalities of Caregiving’, ‘Patient Specific Factors’, ‘Restrictions’ and ‘Caregiver Health’. The qualitative data were quantitated, and the frequency with which they were mentioned is presented in Figure 5. The frequency of themes differed between the three centres. In the Irish cohort, ‘Nothing Difficult’ and ‘Caregiver Psychological/Emotional Distress’ were the most frequently mentioned themes. The Dutch caregivers mentioned ‘Nothing Difficult’ and ‘Practicalities of Caregiving’ most often, and the English caregivers most frequently identified ‘Restrictions’ and ‘Caregiver Psychological/Emotional Distress’ as being the most difficult aspect of caregiving. Direct quotations with attributions by national cohort for each theme are presented in Table 3.

## 4. Discussion

Caring for a partner or family member with a neurological disease is recognised as being a source of burden and distress, which often results in lowered levels of quality of life [29]. The caregiver’s physical, mental, and emotional health may influence their ability to provide care [30]. This paper describes the unique and common challenges faced by three cohorts of informal caregivers, which translate into different caregiving experiences. Exploring the caregivers’ subjective assessment of the burden and difficulties experienced provides a more nuanced picture of the complexity of caregiving in this rare disease.

The innovative mixed-methods analysis describes three informal caregiver cohorts in ALS in terms of demographic profile, quality of life, psychological distress, subjective assessment of burden and the difficulties experienced. Our analysis confirms that caregivers in all three centres experience burden and distress and highlights different components of the complexity that is informal caregiving.

In line with previous research [9], most caregivers were women and co-resident spouses/partners of the person with ALS. More son/daughter caregivers were amongst the Irish group, relative to the other centres. The majority of respondents self-assessed their health as excellent-good while also indicating they had long term health problems, e.g., approximately 57% of the English cohort. Overall, 30–40% were in employment at the time of the interview. The average number of hours per week varied considerably between the caregiver cohorts. This could reflect a variety of factors such as the disease stage or levels of patient impairments and requires further detailed study. There are various contextual factors to consider for any future cross-national comparative analysis; however, our exploratory findings point to similarities and differences among the informal caregivers associated with three of the main ALS centres in Europe.

The levels of burden and psychological distress recorded clearly indicate the negative impact on caregiver wellbeing. Test results showed that the mean burden score in the Irish cohort was significantly lower than in the Dutch and English cohorts, and more Irish caregivers were categorised as a low burden. While overall psychological distress was not significantly different across the cohorts, between 39 and 47% of caregivers in all centres were above the cut off (total score ≥ 12) for probable psychological distress [20]. Mean anxiety levels were significantly higher for the Irish and English groups compared to the Dutch group. In terms of quality of life, scores (QOLLTI-F range 0–10) were relatively high for caregivers from the three centres, although there were significant differences between the Irish and Dutch cohorts, the former recording a higher quality of life.

Previous research suggests that patient disease progression impacts caregiver burden and psychological distress, with burden increasing with disease stage [31]. Functional, cognitive, and behavioural impairment has also been shown to predict caregiver burden [17,32,33,34]. The specific needs of caregivers have been shown to change over time, for example, emotional, social, and professional support [9]. However, some studies demonstrate the limited impact of ALS patient-related variables on caregiver burden and identify the importance of the psychological composition of caregivers [10,33,34]. Therefore, we have more to learn about the factors that affect caregiver burden, but it is likely that the subjective experience of individual caregivers is an important factor influencing the burden experienced.

When asked to describe their difficulties, many caregivers indicated that there were no difficulties, and they were managing well at present. Among the difficulties described by the caregivers in this study, factors associated with ‘psychological/emotional distress’ were mentioned frequently. The responses thematised under ‘psychological/emotional distress’ included *fear for the future*, *anger*, *frustration*, *sadness* and *the emotional impact of watching someone suffer*. *Intruding into patient’s privacy* and *hindering patient autonomy* were aspects expressed by English caregivers only.

The difficulties in relation to the ‘practicalities of caregiving tasks, such as *reduced mobility*, *falls*, and *personal care* identified as contributors to carer burden, were clear. The tasks that needed to be done impacted the ‘caregiver’s own health’. There was the *physical toll of lifting* and carrying bodies with reduced mobility, *difficulties with aids and appliance*, and *coping with behaviour and mood disturbances* as patients refused outside assistance and were considered unappreciative. Difficulties associated with ‘restrictions’ on *time* and ‘limitation’ on *personal and social interaction* were identified. A sense of their own *lives being constrained, being depended upon*, and feeling the *pressure of extra responsibilities*. The Irish and Dutch cohorts mentioned *nothing being difficult at present*. For the Dutch respondents, the practical aspects of care were identified as difficult but psychological/emotional distress less so. English caregivers mentioned difficulties impacting their own health, restrictions, and aspects of psychological/emotional distress frequently. Difficulties associated with psychological/emotional distress were among those often identified by the Irish respondents.

The qualitative responses should be considered in the context of the quantitative wellbeing scores. Anxiety, burden, and quality of life outcomes are given some context by the qualitative responses and frequency with which themes were mentioned by respondents from each centre. The descriptions of the difficulties associated with caregiving suggest specific needs of caregivers in each cohort. Identifying similarities and differences in the themes expressed provide insights into the specific needs of caregivers. This will enable the provision of better informed and more effective interventions for informal caregivers. For example, by understanding that psychological/emotional distress is a common difficulty, we can strive to provide routine psychological evaluations and support to caregivers during the course of the disease. Providing these informed interventions to caregivers may address some of the complexities of the difficulties associated with caregiving and may help clinicians concerned about the caregiver and patient in knowing which type of supports are required. Overall, this enhances the care of the person with ALS.

This was an exploratory analysis of the burden experiences of informal caregivers; further analyses will be considered if experiences vary by the relationship with the patient, duration and severity of illness, and presence of patient cognitive/behavioural impairment. Cross-national comparisons, which factor in contextual factors and health system configurations, are required. In addition, a series of focus groups are planned as a follow-up to explore some of the issues raised in this analysis in greater detail.

## 5. Conclusions

In ALS, where treatment is palliative, and most people live and die at home, informal care is an essential part of the healthcare ecosystem. This descriptive exploratory analysis of caregivers of people with ALS attending three European multidisciplinary clinical centres has described the burden, psychological, and emotional impacts associated with informal care. A holistic understanding of caregiver wellbeing is important to best support the caregiver and enable optimal informal care in progressive, palliative conditions such as ALS.

## Figures and Tables

**Table 1:** Caregiver Characteristics.

Characteristics	ALS Centre			*p-*Value
	Dublin (*n* = 76)	Utrecht (*n* = 58)	Sheffield (*n* = 38)	
**Caregiver Age (years)**				
Mean (SD)	57.03 (13.75)	65.41 (7.88)	60.01 (9.54)	<0.0005 *
Median (IQR)	58.52 (18.63)	66.05 (9.82)	62.13 (12.82)	
Range	26.78–80.84	44.74–80.13	39.88–74.01	
**Sex *N* (%)**				0.100
Male	20 (26.3%)	21 (38.2%)	7 (18.4%)	
Female	56 (73.7%)	34 (61.8%)	31 (81.6%)	
**Relationship to patient *N* (%)**				0.229
Spouse/partner	60 (78.9%)	49 (89.1%)	32 (84.2%)	
Son/daughter	10 (13.2%)	2 (3.6%)	2 (5.3%)	
Parent	2 (2.6%)	-	3 (7.9%)	
Sibling	2 (2.6%)	2 (3.6%)	-	
Other ^a^	2 (2.6%)	2 (3.6%)	1 (2.6%)	
**Living with patient *N* (%)**				1.000
Yes	66 (88%)	49 (89.1%)	34 (89.5%)	
No	9 (12%)	6 (10.9%)	4 (10.5%)	
**Current employment status *N* (%)**				0.231
Employed	31 (43.1%)	18 (32.7%)	12 (32.4%)	
Retired	24 (33.3%)	27 (49.1%)	20 (54.1%)	
**Hours of care provided to patient (p/w)**				
Mean	35.11	96.86	71.08	
Median	9.50	142.5	30	<0.0005 *
Range	0–168	1–168	0–168	
**Health status *N* (%)**				0.003 *
Excellent	18 (24.3%)	8 (14.5%)	4 (10.8%)	
Very good	21 (28.4%)	7 (12.7%)	18 (48.6%)	
Good	24 (32.4%)	32 (58.2%)	9 (24.3%)	
Fair	8 (10.8%)	7 (12.7%)	4 (10.8%)	
Poor	3 (4.1%)	1 (1.8%)	2 (5.4%)	
**Long term illness, health problems, or disability *N* (%)**				0.104
Yes	24 (41.4%)	19 (34.5%)	21 (56.8%)	
No	34 (58.6%)	36 (65.5%)	16 (43.2%)	

Some caregivers did not complete all questions and measures. ^a^ Includes aunt, brother-in-law, stepson, and neighbours. * The mean difference is significant at the 0.05 level. Bold to indicate statistical significance.

**Table 2:** Caregiver Wellbeing Outcomes.

	ALS Centre			*p-*Value
Outcome Measures	Dublin (*n* = 76)	Utrecht (*n* = 58)	Sheffield (*n* = 38)	
**Burden**	*n* = 71	*n* = 53	*n* = 34	
**ZBI Total**				
Mean (SD)	14.7 (11.28)	23.17 (13.57)	24.47 (14.21)	**<0.001** *
Median (IQR)	11 (16)	23 (19.5)	23.5 (19)	
Min, Max	0, 52	1, 56	4, 58	
Shapiro-Wilk *p*-value	0.000	0.098	0.128	
High Burden ^a^ *N* (%)	15 (21.2%)	26 (49.1%)	17 (50%)	0.001 *
Low Burden ^a^ *N* (%)	56 (78.9%)	27 (50.9%)	17 (50%)	
**Psychological Distress**	*n* = 72	*n* = 54	*n* = 34	
**HADS-T**				
Mean (SD)	12.74 (8.59)	10.76 (6.91)	11.88 (8.06)	0.607
Median (IQR)	10 (12.75)	9 (9)	11 (9.25)	
Min, Max	0, 32	0, 31	1, 31	
Shapiro-Wilk *p*-value	0.001	0.006	0.019	
Probable Distress ^b^	31 (43.1.6%)	21 (38.9%)	16 (47.1%)	0.746
**HADS-A**				
Mean (SD)	7.86 (4.65)	5.85 (3.4)	7.44 (4.63)	0.016 *
Median (IQR)	8 (7)	5.5 (4.25)	7 (5.25)	
Min, Max	0, 18	0, 14	0, 19	
Shapiro-Wilk *p*-value	0.074	0.122	0.093	
**HADS-D**				
Mean (SD)	4.88 (4.41)	4.91 (4.05)	4.44 (3.73)	0.860
Median (IQR)	4 (6.75)	3 (6)	4 (5.25)	
Min, Max	0, 16	0, 17	0, 13	
Shapiro-Wilk *p*-value	0.000	0.000	0.005	
**Quality of Life**	*n* = 69	*n* = 45	*n* = 33	
**QOLLTI-F Total**				
Mean (SD)	7.22 (1.38)	6.51 (1.08)	6.91(1.27)	0.009 *
Median (IQR)	7.39 (1.76)	6.55 (1.94)	6.94 (1.59)	
Min, Max	3.68, 9.29	4.54, 8.4	4.22, 9.14	
Shapiro-Wilk *p*-value	0.019	0.147	0.272	

Not all respondents completed all measures. * The mean difference is significant at the 0.05 level. Bold to indicate statistical significance. ^a^ Cut-off score of ≥ 24 for High Burden [23]. ^b^ Cut-off score of ≥12 for probable psychological distress [20].

**Table 3:** Difficulties described, themes, and illustrative quotes.

Themes & Subthemes	Quotes
**Caregiver Psychological/Emotional** **Distress**	
Watching the patient suffer or deteriorate.	“Seeing my wife gradually deteriorate and knowing where it is going makes me sad and slightly afraid of a future without her.”—**English** “Pulling at her shoulders and don’t know if you’re hurting her or not. Getting her into the car is difficult—have to push her in don’t know if hurt shoulder.”—**Irish** “for a loved one to be unable to do everyday tasks.”—**English**“watching him suffer … when he coughs.”—**Irish** “Emotions. He cries a lot. I hate to see that.”—**Irish** “Every time when things deteriorate it is always tough. Injecting insulin is also difficult, how will that be done later or with eating?”—**Dutch** “Seeing this wonderfully active, dignified, clever man being reduced in every way. Communication.”—**English**
Concern for patient privacy and independence.	“Trying not to take away my partners dignity. Trying not to step in too quickly and taking his independence with tasks prematurely.”—**English** “That my husband still feels he can do all of the same things and keeps doing them even though that means he sometimes suffers as a result later. Wanting to help but not making it obvious so that he doesn’t feel any worse than he already does.”—**English**
Anger, sadness, worry, stress, fear, frustration, uncertainty, and guilt.	“Difficult are the things that you can’t change. Also, the fact that there is no chance of a cure is unacceptable. It is very hard to find a way of how to cope with that.”—**Dutch** “I hate to see him helpless.”—**Irish** “Mental support concerning fear of death.”—**Dutch** “The fear of what’s going to come.”—**Irish**
**Practicalities of Caregiving**	
Practicalities related to washing, mobility, managing aids and interventions, household tasks, personal care.	“Everyday life is more difficult.”—**English** “At first it was difficult, for example working with the hoist?”—**Dutch** “Not having the information to ensure you are giving or arranging the correct care so that life is more controllable for him, and he can continue doing things that he does do.”—**Irish** “The only thing that is difficult is when my husband falls down and I can’t pick him up by myself.”—**Dutch** “Transport, not being a driver having to depend on family and friends.”—**English** “The showering is physical/manual.”—**Irish**
**Patient-Specific Factors**	
Patient behavioural factors, disease related.	“Sometimes my wife has a hard time to accept my help, she wants to do more herself, but I think that is not good for her.”—**Dutch** “He won’t let you get someone to do things wants to do it himself.”—**Irish**
	“when talking becomes impaired.”—**Dutch** “It if came to the stage of lifting I don’t know if I could manage.”—**Irish** “Transfers, physically.”—**Irish**
**Restrictions and Limitations**	
Time restrictions, confined to the home, less social activities, more responsibility, having less privacy, dependency of patient on caregiver, relationship changes.	“It is the tie, the responsibility, having to be here.”—**Irish** “Not being able to do something spontaneously everything has to be arranged.”—**Dutch** “Feeling sad as I am his mum. Should be the other way around. Want to give his life back but I can’t. Frustrating, tiring.”—**English** “Everything is more time consuming. Lots of appointments exhausting and take up family time. Going out, travelling is more difficult. We have less money, and everything is more expensive.”—**English** “Not being able to lead my own life. To be under pressure, there is always something that need to be done. You can’t postpone. Everything HAS TO BE done. This pressure has been imposed on us because this happened to us. We did not choose this. ourselves. That feels like a burden.”—**Dutch** “Everyone was coming to the house. Always someone here. When I came home from work. Didn’t have time for us to talk. Had no space. At the time of diagnosis. Better now. No privacy, can’t sit down and talk ourselves.”—**Irish** “reversed roles between parent and child.”—**Dutch** “Working everything around my mum’s needs/appointments.”—**English**
**Caregiver Health**	
Physical health and ability to undertake tasks.	“My back is definitely not good and my knees. Lack of sleep.”—**Irish** “My own physical strength, and a tendency to “putting my back out” will be a problem.”—**English** “Lack of sleep.”—**English** “In a phase of general fatigue.”—**Dutch**
**Nothing difficult**	“There’s no difficulty.”—**English** “nothing difficult about it now.”—**Irish** “nothing is difficult, everything is automatic in terms of medical aids.”—**Dutch** “, there isn’t any caregiving at all. She is well able to take care of herself.”—**Irish**
**Other**	
Including just getting on with it, lacking information, not wanting outside help, caring for others besides the patient, and having less money.	“not really relevant at the moment.”—**Irish** “little care is needed for the patient at the moment.”—**Dutch**

## Data Availability

The data presented in this study are available upon reasonable individual request to Mark Heverin, Research Manager in the Academic Unit of Neurology in Trinity College Dublin.

## References

[1] Rooney J., Byrne S., Heverin M., Corr B., Elamin M., Staines A., Goldacre B., Hardiman O. (2013). Survival Analysis of Irish Amyotrophic Lateral Sclerosis Patients Diagnosed from 1995–2010. PLoS ONE.

[2] Connolly S., Galvin M., Hardiman O. (2015). End-of-life management in patients with amyotrophic lateral sclerosis. Lancet Neurol..

[3] Galvin M., Gavin T., Mays I., Heverin M., Hardiman O. (2020). Individual quality of life in spousal ALS patient-caregiver dyads. Health Qual. Life Outcomes.

[4] de Wit J., Bakker L.A., van Groenestijn A.C., van den Berg L.H., Schröder C.D., Visser-Meily J.M.A., Beelen A. (2018). Caregiver burden in amyotrophic lateral sclerosis: A systematic review. Palliat. Med..

[5] Kaub-Wittemer D., Von Steinbüchel N., Wasner M., Laier-Groeneveld G., Borasio G.D. (2003). Quality of life and psychosocial issues in ventilated patients with amyotrophic lateral sclerosis and their caregivers. J. Pain Symptom Manag..

[6] Trail M., Nelson N.D., Van J.N., Appel S.H., Lai E.C. (2003). A study comparing patients with amyotrophic lateral sclerosis and their caregivers on measures of quality of life, depression, and their attitudes toward treatment options. J. Neurol. Sci..

[7] Aoun S.M., Bentley B., Funk L., Toye C., Grande G., Stajduhar K. (2013). A 10-year literature review of family caregiving for motor neurone disease: Moving from caregiver burden studies to palliative care interventions. Palliat. Med..

[8] Baxter S.K., Baird W.O., Thompson S., Bianchi S.M., Walters S.J., Lee E., Ahmedzai S.H., Proctor A., Shaw P., McDermott C.J. (2013). The Impact on the Family Carer of Motor Neurone Disease and Intervention with Noninvasive Ventilation. J. Palliat. Med..

[9] Galvin M., Carney S., Corr B., Mays I., Pender N., Hardiman O. (2018). Needs of informal caregivers across the caregiving course in amyotrophic lateral sclerosis: A qualitative analysis. BMJ Open.

[10] Burke T., Galvin M., Pinto-Grau M., Lonergan K., Madden C., Mays I., Carney S., Hardiman O., Pender N. (2017). Caregivers of patients with amyotrophic lateral sclerosis: Investigating quality of life, caregiver burden, service engagement, and patient survival. J. Neurol..

[11] Association MND (2019). Caring for Carers of People with MND: How Government Can Help. Motor Neurone Disease Association. https://www.mndassociation.org/app/uploads/2019/02/MND_Association_report_Carers_Strategy.pdf.

[12] Ryan M., Heverin M., McLaughlin R., Hardiman O. (2019). Lifetime Risk and Heritability of Amyotrophic Lateral Sclerosis. JAMA Neurol..

[13] Gowland A., Opie-Martin S., Scott K.M., Jones A.R., Mehta P., Batts C.J., Ellis C.L., Leigh P.N., Shaw C.E., Sreedharan J. (2019). Predicting the future of ALS: The impact of demographic change and potential new treatments on the prevalence of ALS in the United Kingdom, 2020–2116. Amyotroph. Lateral Scler. Front. Degener..

[14] Galvin M., Gaffney R., Corr B., Mays I., Hardiman O. (2017). From first symptoms to diagnosis of amyotrophic lateral sclerosis: Perspectives of an Irish informal caregiver cohort—A thematic analysis. BMJ Open.

[15] de Jongh A.D., van Eijk R.P.A., Peters S.M., van Es M.A., Horemans A.M.C., van der Kooi A.J., Voermans N.C., Vermeulen R.C.H., Veldink J.H., van den Berg L.H. (2021). Incidence, Prevalence, and Geographical Clustering of Motor Neuron Disease in the Netherlands. Neurology.

[16] A Programme for Amyotrophic Lateral Sclerosis Care in Europe (ALS-CarE). https://clinicaltrials.gov/ct2/show/NCT03081338.

[17] Burke T., Elamin M., Galvin M., Hardiman O., Pender N. (2015). Caregiver burden in amyotrophic lateral sclerosis: A cross-sectional investigation of predictors. J. Neurol..

[18] Zigmond A.S., Snaith R.P. (1983). The Hospital Anxiety and Depression Scale. Acta Psychiatr. Scand..

[19] Norton S., Cosco T., Doyle F., Done J., Sacker A. (2013). The Hospital Anxiety and Depression Scale: A meta confirmatory factor analysis. J. Psychosom. Res..

[20] Pallant J.F., Tennant A. (2007). An introduction to the Rasch measurement model: An example using the Hospital Anxiety and Depression Scale (HADS). Br. J. Clin. Psychol..

[21] Cohen R., Leis A.M., Kuhl D., Charbonneau C., Ritvo P., Ashbury F.D. (2006). QOLLTI-F: Measuring family carer quality of life. Palliat. Med..

[22] Zarit S.H., Reever K.E., Bach-Peterson J. (1980). Relatives of the impaired elderly: Correlates of feelings of burden. Gerontologist.

[23] Schreiner A.S., Morimoto T., Arai Y., Zarit S. (2006). Assessing family caregiver’s mental health using a statistically derived cut-off score for the Zarit Burden Interview. Aging Ment. Health.

[24] Morgan D.L. (2014). Integrating Qualitative and Quantitative Methods: A Pragmatic Approach.

[25] (2019). IBM SPSS Statistics for Macintosh.

[26] Braun V., Clarke V., Cooper H., Camic P.M., Long D.L., Panter A.T., Rindskpf D., Sher K.J. (2012). Thematic analysis. APA Handbook of Research Methods in Psychology, Vol. 2. Research Designs: Quantitative, Qualitative, Neuropsychological, and Biological.

[27] Miles M.B., Huberman A.M. (1994). Qualitative Data Analysis: An Expanded Sourcebook.

[28] O’Connor E.J., McCabe M.P. (2010). Predictors of quality of life in carers for people with a progressive neurological illness: A longitudinal study. Qual. Life Res..

[29] Schulz R., Sherwood P.R. (2008). Physical and mental health effects of family caregiving. Am. J. Nurs..

[30] Chiò A., Hammond E.R., Mora G., Bonito V., Filippini G. (2015). Development and evaluation of a clinical staging system for amyotrophic lateral sclerosis. J. Neurol. Neurosurg. Psychiatry.

[31] Caga J., Hsieh S., Lillo P., Dudley K., Mioshi E. (2019). The Impact of Cognitive and Behavioral Symptoms on ALS Patients and Their Caregivers. Front. Neurol..

[32] Schischlevskij P., Cordts I., Günther R., Stolte B., Zeller D., Schröter C., Weyen U., Regensburger M., Wolf J., Schneider I. (2021). Informal Caregiving in Amyotrophic Lateral Sclerosis (ALS): A High Caregiver Burden and Drastic Consequences on Caregivers’ Lives. Brain Sci..

[33] Cui B., Cui L., Gao J., Liu M., Li X., Liu C., Ma J., Fang J. (2015). Cognitive Impairment in Chinese Patients with Sporadic Amyotrophic Lateral Sclerosis. PLoS ONE.

[34] Galvin M., Corr B., Madden C., Mays I., McQuillan R., Timonen V., Staines A., Hardiman O. (2016). Caregiving in ALS—A mixed methods approach to the study of Burden. BMC Palliat. Care.

